# Which Bayley-III cut-off values should be used in different developmental levels?

**DOI:** 10.3906/sag-1910-69

**Published:** 2020-06-23

**Authors:** Pelin ÇELİK, İclal AYRANCI SUCAKLI, Halil İbrahim YAKUT

**Affiliations:** 1 Department of Pediatrics, Division of Developmental and Behavioral Pediatrics, Ankara City Hospital, Ankara Turkey; 2 Department of Pediatrics, Ankara City Hospital, Ankara Turkey

**Keywords:** BSID-II, Bayley-III, 6–42 months of age, developmental delay levels, cut-off scores

## Abstract

**Background/aim:**

Latest version of Bayley Scales (Bayley-III) and its predecessor (BSID-II) are the most widely used standardized developmental tools in infancy and early childhood. Recent studies showed that Bayley-III scores were higher than BSID-II in 18–24 month-old and mostly premature infants. We aimed to evaluate the generalization of inflated scores of Bayley-III to children aged 6–42 months with different disease groups, and to find out which cut-off points should be used in Bayley-III to detect mild, moderate, and severe developmental delay according to BSID-II standard cut-off points.

**Materials and methods:**

Two hundred and fifty-five children aged 6–42 months with different diseases and developmental levels were administered both the Bayley-III and BSID-II in the same session between 15 November 2017 and 15 April 2018.

**Results:**

The mean Bayley-III Cognitive Composite (CC) and Cognitive Language Composite (CLC) scores were respectively 13.1 ± 9.1 and 8.6 ± 8 points higher than BSID-II Mental Development Index (MDI) scores (P < 0.001). The mean Bayley-III Motor Composite (MC) scores were 14.4 ± 10.5 points higher than BSID-II Psychomotor Developmental Index (PDI) scores (P < 0.001). Cognitive delay was found in 126 (49.4%) and 59 (23.1%) children according to BSID-II MDI and Bayley-III CC scores, respectively. Motor delay was found in 174 (69.3%) and 86 (34.3%) children according to the BSID-II PDI and Bayley-III MC scores, respectively. Children had less cognitive (48.6%) and motor delay (54.5%) according to Bayley-III scores. Bayley-III scores were significantly higher than BSID-II scores for all ages (P < 0.001). According to ROC analysis the cut-off scores for mild, moderate, and severe delay were 92.5, 83.2, and 71.2 for Bayley-III CLC; and 98.5, 86.5, and 74.5 for Bayley-III MC, respectively.

**Conclusion:**

Bayley-III scores should be interpreted carefully for all age ranges and different diagnosis. The risk for underestimation of developmental delays by Bayley-III should be kept in mind. Different Bayley-III cut-off scores should be used to define developmental delay levels.

## 1. Introduction

Bayley Scales of Infant and Toddler Development 2nd Edition (BSID-II) and its latest version Bayley Scales of Infant and Toddler Development 3rd Edition (Bayley-III) are currently the most widely used standardized developmental tools in both clinical practice and research settings for assessment of development in infancy and early childhood (0–42 monhts), early diagnosis of developmental delays, providing information for early intervention planning, and assessment of the efficiency of these interventions.

The Bayley Scales of Infant Development was first published in 1969 [1], it was updated and standardized in 1993 as BSID-II [2]. BSID-II is comprised of two scales, the Mental Developmental Index (MDI) and the Psychomotor Developmental Index (PDI). MDI measures the combination of the nonverbal cognitive and language skills, and PDI measures the combination of fine and gross motor skills. The third edition, Bayley-III, was published in 2006, and MDI was divided into cognitive, receptive language, and expressive language subscales, and the PDI into fine motor skills and gross motor skills subscales [3]. Thus, Bayley-III provides significant advantages over BSID-II with regard to assessing the cognitive, receptive language, expressive language, fine motor, and gross motor skills of the child separately and offers more detailed and clear information about areas that may benefit from targeted interventions.

The concerns have risen gradually since 2010 that scores on Bayley-III are higher than those obtained with the BSID-II, and Bayley-III may identify significantly fewer children with developmental delay compared to the BSID-II [4-7]. These studies were predominantly conducted in premature infants and specific age ranges such as between 18 and 24 months. No study has been conducted so far about whether Bayley-III’s higher scores for neurodevelopment than BSID-II can be generalization to 0–42 months age range and to infants with different diseases. Based on the standard cut-off scores of 85, 70, and 55 of BSID-II reflecting mild, moderate, and severe developmental delays, studies determining which cut-off points should be used in Bayley-III are limited [8]. Moreover, there is no study comparing BSID-II and Bayley-III in Turkish children.

We aimed to evaluate the generalization of inflated scores of Bayley-III to children aged 6–42 months and also with different disease groups, and to find out which cut-off points should be used in Bayley-III to detect mild, moderate, and severe developmental delay according to BSID-II standard cut-off points.

## 2. Materials and methods

### 2.1. Study design and participants

This prospective study was conducted in Ankara Child Health and Diseases Hematology and Oncology Training and Research Hospital, University of Health Sciences Turkey. Ethical committee of approval was obtained from Etlik Zübeyde Hanım Women’s Health Teaching and Research Hospital, University of Health Sciences Turkey.

Children with developmental risks or difficulties (prematurity, hearing loss, perinatal asphyxia, cerebral palsy, epilepsy, genetic diseases, metabolic diseases, etc.) and healthy children aged 6–42 months admitted to the Developmental-Behavioral Pediatrics Outpatient Clinic between 15 November 2017 and 15 April 2018 were included in the study. Inform consent was taken from parents. 

A A developmental-behavioral pediatrician reviewed the medical records of children, performed detailed neurological and physical examinations, and evaluated their developmental functions, activities, and participation in life.

Bayley-III items were administered first in accordance with its technique. Bayley-III is more detailed and contains more items than BSID-II, and most of the test items in Bayley-III overlap with BSID-II. Thus, Bayley-III and overlapping BSID-II items were coded with the child’s performance. Additional BSID-II items were administered after Bayley-III in the same session. Items were scored according to the instructions of each version. During the assessment, for hearing impaired children, it was ensured that the child was using the hearing aid or cohlear implant correctly; the ambient noise was minimized, and the child was spoken to clearly and naturally. 

The MDI and PDI were calculated from the BSID-II raw scores; and the Cognitive Composite (CC), Language Composite (LC), and Motor Composite (MC) scores were calculated from the Bayley-III raw scores. The Bayley-III Cognitive Language Composite (CLC) was defined as the mean of the CC and LC scores [6,8]. The differences between the BSID-II MDI and Bayley-III CC; BSID-II MDI and Bayley-III CLC; and BSID-II PDI and Bayley-III MC were compared. 

With regard to Bayley scores, mild, moderate, and severe delay were interpreted as <85 points (<-1SD), <70 points (<-2SD), and <55 (<-3SD), respectively. 

Age adjustment was performed in premature infants up to two years. Children who had lost their concentration or could not finish both tests were completely excluded from the study to avoid reporting misleading scores. According to family-centered holistic developmental evaluation, children with developmental difficulties or disabilities were referred to early intervention services. 

### 2.2. Statistical analysis

Statistical analyses were performed using the SPSS statistical package (v. 20.0 for MAC). Categorical variables between groups were analyzed using the χ2 test. Comparison of means between two groups was examined by using a t-test, where the data fit a normal distribution. For comparison of more than two groups, ANOVA was used for normal distributions and the Kruskal–Wallis test for nonnormal distributions. Receiver operating characteristic curve analysis (ROC) was used to determine the power of variables to differentiate groups, and the area under the curve was calculated; significant cut-off levels were calculated using a Youden index. A P value of <0.05 was deemed to indicate statistical significance.

## 3. Results 

A total of 255 children including 115 girls (45.1%) and 140 boys (54.9%) completed both versions of the test. The mean age was 21.1 ± 10.6 months. The mean gestational week at delivery and birth weight were 35.1 ± 5.4 weeks and 2443 ± 1046 g, respectively. The diagnoses of children were prematurity (27.8%), neurological system diseases including cerebral palsy and hypoxic ischemic encephalopathy (18.9%), speech delay (7.8%), genetic diseases (7.5%), hearing loss (6.7%), other diseases (11.4%); and the rest were healthy children (20%). 

The mean BSID-II MDI and PDI scores were respectively 80.7 ± 21.8 and 72.8 ± 20.1. The mean Bayley-III CC, LC, MC, and CLC scores were 93.8 ± 2.1, 84.7 ± 21.9, 87.3 ± 22.2, and 89.2 ± 19.9 respectively. The mean Bayley-III CC and CLC scores were 13.1 ± 9.1 (P < 0.001) and 8.6 ± 8 (P < 0.001) points higher than the BSID-II MDI score respectively. The mean Bayley-III MC score was 14.4 ± 10.5 higher than the BSID-II PDI score (P < 0.001). 

Cognitive delay was found in 126 (49.4%) and 59 (23.1%) children according to BSID-II MDI and Bayley-III CC scores, respectively (Table 1). Of the children, 48.6% (n = 124/255) were classified as less severely cognitive delayed according to the Bayley-III CC scores than BSID-II MDI. Cognitive scores were found <70 in 81 (31.7%) and 38 (14.9%) children according to BSID-II MDI and Bayley-III CC scores, respectively. Motor delay was found in 174 (69.3%) and 86 (34.3%) children according to the BSID-II PDI and Bayley-III MC scores, respectively (Table 2). Of the children, 54.5% (n=137/251) were classified as less severely motor delayed according to the Bayley-III MC scores than BSID-II PDI scores. BSID-II PDI and Bayley-III MC scores were <70 in 116 (46.2%) and 55 (21.9%) children, respectively.

**Table 1:** Bayley-III Cognitive Composite and BSID-II MDI scores.

	BSID-II MDI				
Bayley-III CC	Normal(≥85)	Mild (70–84)	Moderate (55–69)	Severe (<55)	Total, n %
Normal (≥85)	129 (50.6%)	43(16.9%)	20(7.8%)	4(1.6%)	196(76.9%)
Mild (70–84)	0	2(0.8%)	5(2%)	14(5.5%)	21(8.2%)
Moderate (55–69)	0	0	0	38(14.9%)	38(14.9%)
Severe (<55)	0	0	0	0	0
Total	129 (50.6%)	45(17.6%)	25 (9.8%)	56(22%)	255 (100%)

BSID-II, Bayley Scales of Infant and Toddler Development 2nd Edition; Bayley-III, Bayley Scales of Infant and Toddler Development 3rd Edition; CC, Cognitive composite; MDI, Mental Developmental Index

**Table 2:** Bayley-III Motor Composite and BSID-II PDI scores.

	BSID-II PDI				
Bayley-III MC	Normal (≥85)	Mild (70–84)	Moderate (55–69)	Severe (<55)	Total, n %
Normal (≥85)	77 (30.7%)	56(22.3%)	28(11.2%)	4(1.6%)	165(65.7%)
Mild (70–84)	0	2(0.8%)	11(4.4%)	18(7.2%)	31(12.4%)
Moderate (55-69)	0	0	1	20(8%)	21(8.4%)
Severe (<55)	0	0	0	34(13.5%)	34(13.5%)
Total	77 (30.7%)	58(23.1%)	40 (15.9%)	76(30.3%)	251* (100%)

BSID-II, Bayley Scales of Infant and Toddler Development 2nd Edition; Bayley-III, Bayley Scales of Infant and Toddler Development 3rd Edition, MC, Motor Composite; PDI, Psychomotor Developmental Index

When the distribution of children according to age ranges is considered, most of the children were between the ages of 6 and 12 months, 25 and 30 months, and 19 and 24 months, respectively (Table 3). In all age ranges, the mean Bayley-III CC and MC scores were higher than the BSID-II MDI and BSID-II PDI scores (P < 0.001).

**Table 3:** BSID-II index and Bayley-III composite scores according to age ranges.

Age ranges (months)	n (%)	BSID-II MDI	Bayley-III CC	P1	BSID-II PDI	Bayley-III MC	P2
6–12	82 (32.2%)	92.2 ± 19.4	102.6 ± 20	<0.001	76.8 ± 18.2	89.7 ± 20.8	<0.001
13–18	34 (13.4%)	87.2 ± 19.3	98.5 ± 19.2	<0.001	69.4 ± 20.1	85 ± 19.8	<0.001
19–24	41 (16.1%)	74.5 ± 20.2	90.4 ± 19.9	<0.001	77.5 ± 23.3	88.3 ± 25.7	<0.001
25–30	43 (16.9%)	73.4 ± 18.8	92.7 ± 15.8	<0.001	67.7 ± 15.6	90 ± 16.1	<0.001
31–36	26 (10.1%)	70 ± 23.6	79 ± 18.9	<0.001	68.4 ± 22.9	79 ± 28.4	<0.001
37–42	29 (11.4%)	70.8 ± 19.8	83.9 ± 15.2	<0.001	70.3 ± 21.7	84.4 ± 24.2	<0.001

BSID-II, Bayley Scales of Infant and Toddler Development 2nd Edition; Bayley-III, Bayley Scales of Infant and Toddler Development 3rd Edition; CC, Cognitive Composite; MC, Motor Composite; PDI, Psychomotor Developmental Index; MDI, Mental Developmental Index; P1, BSID-II MDI vs Bayley-III CC; P2, BSID-II PDI vs Bayley-III MC

In ROC analysis of a BSID-II MDI score <70, the cut-off value for the Bayley-III CC score was 87.5 (P < 0.001; AUC: 0.97, sensitivity 86.4%, specific­ity 94.1%); 83.2 for the Bayley-III CLC score (P < 0.001; AUC: 0.97, sensitivity 86.4%; specificity 91.1%) (Figure). In ROC analysis of the BSID-II PDI score <70, the cut-off value for the Bayley-III MC score was 86.5 (P < 0.001; AUC: 0.96, sensitivity 77.6%, specific­ity 95.5%). 

**Figure 1:**
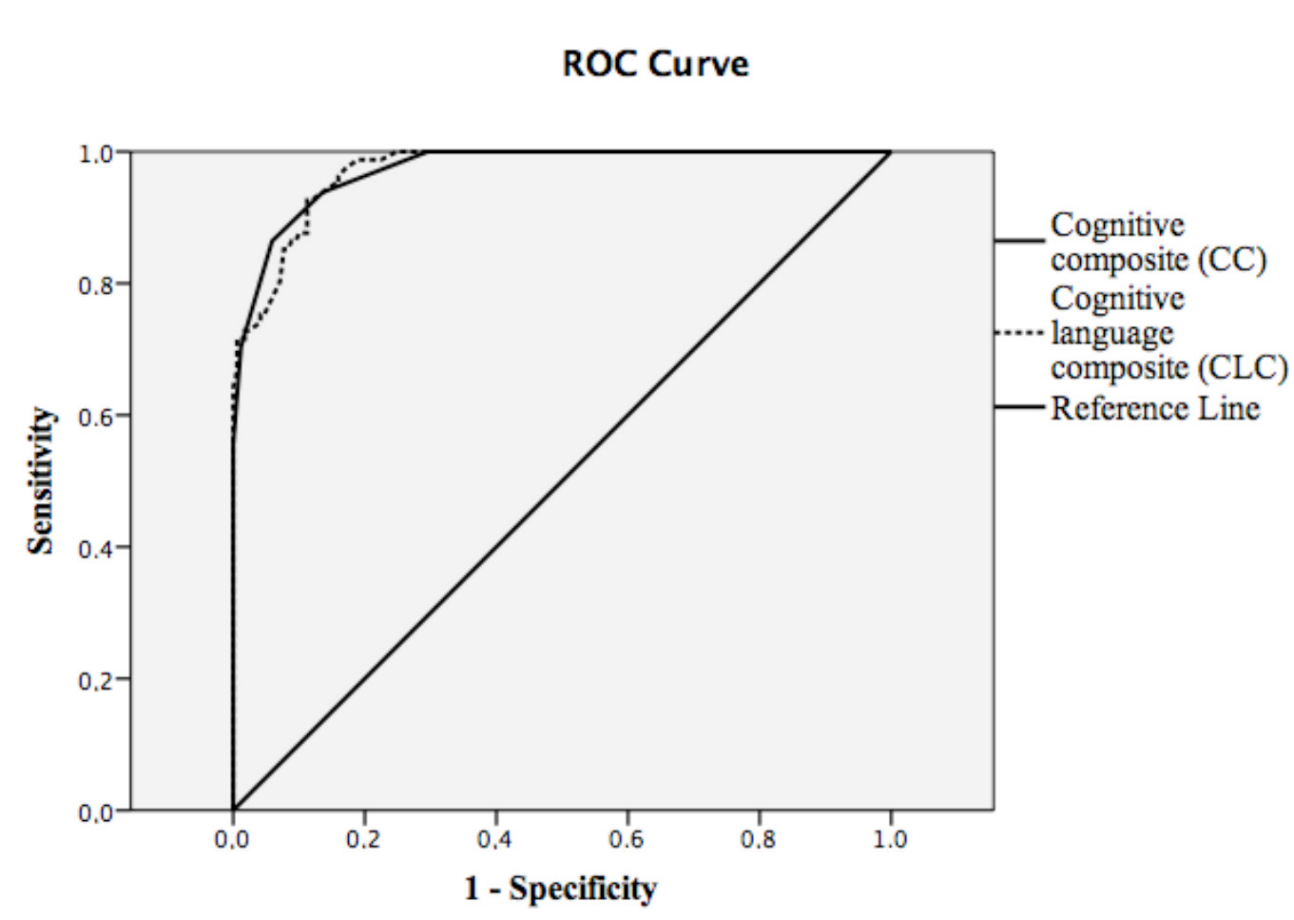
Receiver operating characteristic curve analysis (ROC) of BSID-II MDI score <70 versus Bayley-III CC and Bayley-III CLC scores. BSID-II, Bayley Scales of Infant and Toddler Development 2nd Edition; Bayley-III, Bayley Scales of Infant and Toddler Development 3rd Edition; CC, Cognitive composite; CLC, cognitive language composite; MDI, Mental Developmental Index.

In ROC analysis of a BSID-II MDI score <55, the cut-off values for the Bayley-III CC and Bayley-III CLC scores were 77.5 and 71.2, respectively (P < 0.001; AUC:0.99; sensitivity 85.7%; specificity 98%, and P < 0.001; AUC: 0.99; sensitivity 89.3%; specificity 99%, respectively). In ROC analysis of a BSID-II PDI score <55, the cut-off value for the Bayley-III MC score was 74.5 (P < 0.001; AUC: 0.98; sensitivity 86.8%; specificity 99%). 

In ROC analysis of a BSID-II MDI score <85, the cut-off values for the Bayley-III CC and Bayley-III CLC scores were 97.5 and 92.5, respectively (P < 0.001; AUC: 0.94; sensitivity 89.6%; specificity 98.5%, and P < 0.001; AUC:0.97; sensitivity 91.2%; specificity 92.9%, respectively). (Table 4). In ROC analysis of a BSID-II PDI score <85, the cut-off value for the Bayley-III MC score was 98.5 (P < 0.001; AUC: 0.93; sensitivity 84.5%; specificity 83.1%).

**Table 4:** Ability of different Bayley-III cut-offs to detect BSID-II scores <85.

Test score cut-off	Sensitivity (%)	Specificity (%)
CC score		
<92.5	73.6	94.5
<97.5	89.6	98.5
CLC score		
<87.2	76.8	94.5
<92.5	91.2	92.9
MC score		
<92.5	70.7	94.8
<98.5	84.5	83.1

BSID-II, Bayley Scales of Infant and Toddler Development 2nd Edition; Bayley-III, Bayley Scales of Infant and Toddler Development 3rd Edition; CC, Cognitive Composite; CLC, Cognitive Language Composite; MC, Motor Composite

## 4. Discussion

In our study, mean Bayley-III CC and MC scores of the children at 6–42 months age range with different medical diagnoses and different developmental levels were found to be almost 1 SD higher (13.1 and 14.4 points, respectively) than the mean BSID-II MDI and PDI scores. CLC scores were 8.6 ± 8 points higher than the BSID-II MDI score. In previous studies conducted with different methodologies, mean Bayley-III CC and CLC scores were reported to be 6–14 points [5,6,8–13] and 5.8–7 points [8,9] higher than the MDI scores; and mean Bayley-III MC scores were 6.9–18 points higher than the PDI scores [5,6,8–13]. We also found similar differences between Bayley-III and BSID-II scores.

While most of the studies were performed in preterm infants [4,5,7,9,12–17], only two studies were conducted with children with different diagnoses such as neonatal encephalopathy [6] and complex cardiac surgery history [10]. Our study was performed in children with different medical diagnoses and different developmental levels. Studies were commonly performed in children aged 18–24 months [4–6,10,12–16] whereas other age ranges such as our study were rarely studied [9,11,15,17]. Our study included children at the 6–42 months age group to find out whether Bayley-III overestimate the developmental status in different age groups or not. The mean Bayley-III scores in our study were significantly higher than the BSID-II scores in all age ranges through 6–42 months. Our findings indicate that the overestimation of the development by Bayley-III could be generalized to children at the 6–42 months age range.

It was reported that 40% and 48.1% of the children were classified as less severely cognitive and motor delayed with the Bayley-III scores than with the BSID-II [5,12]. In our study, the levels of cognitive delay and motor development delay were classified as less severely delayed in 48.6% and 54.5% of the children using the Bayley-III compared to BSID-II. The discrepancy between BSID-II and Bayley-III in determining the level of developmental delay was higher in our study compared to previous studies. This might be related to the fact that our study was performed in children with different age ranges, diagnoses, and developmental levels.

In some studies new cut-off points for Bayley-III were determined to correct inflated scores, classify the developmental delays accurately, and to interpret the results of the studies correctly because Bayley-III has widespread use and no alternative. Studies suggested taking <80–85 as a Bayley-III CC score cut-off for the <70 BSID-II MDI score [5–7,9] ; and in one study the cut-off value was reported as <93 points [14]. In another study, <80 points was indicated as the best cut-off point for Bayley-III CLC score corresponding to the <70 BSID-II MDI score, and it was argued that Bayley-III CLC scores had the advantage of producing a single continuous outcome; however, it required more confirmation [7]. 

The studies that define the optimal cut-off value for Bayley-III MC are limited. Jary et al. suggested the optimal Bayley-III MC cut-off for the identification of BSID-II PDI <70 was <85 [6] whereas Duncan et al. suggested <73 as cut-off in their study conducted using the National Institute of Child Health and Human Development Neonatal Research Network data [16]. In another study, it was asserted that the Bayley-III MC cut-off composite scores should be 12–24 points higher than 70 for optimal prediction of the motor delay as defined by the BSID-II index score <70 [15].

However, most of the previous studies focused on the cut-off value for moderate developmental delay (70 points); there is only one study identifying optimal cut-off values for mild (85 points) and severe (55 points) developmental delay in 62 children [8]. They suggested to use 87.3, 78.0, and 67.0 in Bayley-III CLC instead of 85, 70, and 55 in the BSID-II MDI respectively, for mild, moderate, and severe cognitive delays [8]. In the same study, it was suggested that the scores should be increased from 70 to 80 and from 55 to 68.5 for the moderate and severe motor delay, respectively [8]. In our study, it was found that 92.5, 83.2, and 71.2 cut-off points should be used in the Bayley-III CLC score; and 98.5, 86.5, and 74.5 cut-off points should be used in the Bayley-III MC score respectively for the mild, moderate, and severe delays. We found higher cut-off levels than Yi et al. This may be associated with higher number of participants in our study and our study included healthy children and children with different developmental levels.

It was shown that the difference between the BSID-II and Bayley-III scores was not linear, and the gap between the two scales increased as the severity of the developmental delay increased [8,9]. Similarly we found that the discrepancy between the BSID-II and Bayley-III scores was 7.5, 13.2, and 16.2 points for the mild, moderate, and severe cognitive delay respectively; and 13.5, 16.5, and 19.5 points for the mild, moderate, and severe motor developmental delay, respectively. The relatively higher Bayley-III scores are important in terms of inadequate detection of children with developmental delay and need for intervention services. The largest gap between the two scales at the lowest end of the scores might cause the children who may benefit most from early intervention services to be detected as not requiring these services, and leads them not to receive services. Moreover, as Bayley-III is a widely used tool in research, relatively higher scores may lead to inadequate detection of the prevalence and severity of developmental delays in clinical populations.

The standardization of Bayley II was performed in children who were completely healthy. Bayley-III was normed using a “mixed sampling procedure”. In other words, approximately 10% of the standardization sample consists of children with developmental difficulties or delay such as Down syndrome, cerebral palsy, pervasive developmental disorder, prematurity, and speech delay. This mixed sampling procedure is likely to lead to a decrease in the raw scores, which would constitute the standard average score of 100, the increase in standard deviation, and the expected performance from the normative population, and to decrease the capacity to identify developmental delay [18]. This might be the reason for the increasing discrepancy between the two tests. In fact, expected performances for the common test items that measure the same developmental function in both versions of the test are scored at the older age range in Bayley-III and this may support the difference between Bayley-III and BSID-II. For example, while the performance in the test item “completes correctly pink board within 180 s” is expected at 23–25 months age range in BSID-II, it is expected at the 25 months 16 days–28 months 15 days age range in Bayley-III. While the “uses a two-word utterance” performance is expected at the 23–25 months age range in BSID-II, it is expected at the 28 months 16 days–32 months 30 days age range in Bayley-III. There are numerous examples of similar test items. 

There are few studies about prediction of later functioning by Bayley-III and BSID-II. In a meta-analysis study, a strong correlation between the MDI scores and cognitive functioning at later ages, and a weak correlation between the PDI scores and the motor functioning at later ages were found (P < 0.001) [19]. Different results including high [20] and low [21] predictivity were reported about the value of 2-year-old Bayley-III cognitive and language composite scores in predicting cognitive functioning at the age of 4. It was stated that Bayley-III motor scores had a high specificity in predicting later motor impairment with a low sensitivity, and Bayley-III was less able to detect later motor impairment [22]. We will follow our patients to evaluate which scale will predict later functioning better.

Our research is the first study to show that the mean Bayley-III scores were higher than the BSID-II scores in all age ranges at 6–42 months aged children. In addition, it is a strong aspect of our study that it included children with different diagnoses and developmental levels. This is the largest study to date that determined cut-off values for mild and severe developmental delays, in addition to moderate delay for Bayley-III. It was also the first study in the Turkish population to compare the two scales.

Although fatigue cases are excluded, both versions of the tests were performed in the same session, which might have resulted in assessment bias that led to inflated test scores. The administering of BSID-II and Bayley-III by the same specialist is another limitation of our study. 

In conclusion, Bayley-III scores should be interpreted carefully for all age ranges and different diagnoses. The risk for underestimation of developmental delays by Bayley-III should be kept in mind. Different Bayley-III cut-off values from BSID-II should be used to define developmental delay levels. Long-term results are needed to determine which scale has better predictive value for later cognitive and motor functioning.
